# Netrin‐1 and B‐cell maturation antigen expression in a large cohort of 361 lymphomas: sensitive and specific staining in plasmablastic lymphomas, and therapeutic perspectives

**DOI:** 10.1002/2056-4538.70027

**Published:** 2025-04-15

**Authors:** Marie Donzel, Alexis Trecourt, Hervé Ghesquières, Thi‐Thuy‐Trinh Nguyen, Sara Dandash, Morgane Denis, Emeline Cros‐Perrial, Juliette Fontaine, Charles Dumontet, Alexandra Traverse‐Glehen

**Affiliations:** ^1^ Hospices Civils de Lyon Institut de pathologie multisite, Hôpital Lyon Sud Lyon France; ^2^ Université Claude Bernard Lyon 1 Lyon France; ^3^ Centre International de Recherche en Infectiologie (CIRI) Institut national de la santé et de la recherche médicale (INSERM) U1111, Centre national de la recherche scientifique (CNRS), UMR5308, Ecole normale supérieure de Lyon Lyon France; ^4^ Hospices Civils de Lyon, service d'hématologie Hôpital Lyon Sud Lyon France; ^5^ Cancer Research Center of Lyon (CRCL) INSERM 1052/CNRS 5286/University of Lyon Lyon France

**Keywords:** BCMA, Netrin‐1, immunocytochemistry, lymphoma, plasmablastic, lymphoma, classical Hodgkin lymphoma

## Abstract

Netrin‐1 and B‐cell maturation antigen (BCMA) are currently being evaluated as therapeutic targets in oncology. However, studies investigating their expression in mature human lymphoid malignancies are sparse. This study aimed to investigate the expression of BCMA and Netrin‐1 in a large cohort of lymphomas to determine their potential role as biomarkers or therapeutic targets. BCMA and Netrin‐1 expression was investigated comprehensively using immunohistochemistry in a cohort that included 261 B‐cell lymphomas, 45 T‐cell lymphomas, and 55 classical Hodgkin lymphomas. Netrin‐1 displayed a cytoplasmic staining pattern in plasmablastic lymphomas (27/28, 96%) and classical Hodgkin lymphomas (8/55, 15%). BCMA displayed cytoplasmic staining in most plasmablastic lymphomas (17/20, 85%). Among mature B‐cell lymphomas, Netrin‐1 and BCMA displayed sensitive (96% and 85%, respectively) and specific (100% and 95%, respectively) staining in plasmablastic lymphomas. These results suggest that these proteins may help pathologists in complex diagnoses and reinforce the interest in developing clinical trials assessing Netrin‐1 or BCMA‐targeted therapies in plasmablastic lymphoma and classical Hodgkin lymphomas, for which our therapeutic arsenal is weak.

## Introduction

Netrin‐1 is a protein encoded by the Netrin‐1 gene (*NTN1*), a member of a family of laminin‐related secreted proteins, which is involved in axon guidance and cell migration during development [1, 2]. B‐cell maturation antigen (BCMA) is a transmembrane receptor belonging to the tumor necrosis factor receptor superfamily, which is involved in B‐cell maturation and differentiation into plasma cells [3, 4]. These proteins are currently being evaluated as therapeutic targets in oncology: (1) in the field of solid tumors for Netrin‐1, which is thought to be involved in the tumorigenesis of numerous carcinomas [1, 5, 6, 7]; and (2) in multiple myeloma (MM) for BCMA [3]. Therapeutic perspectives emerged from these studies [2, 3, 8, 9], but further studies are required to clarify the role of Netrin‐1 and BCMA in mature lymphoid neoplasms.

Studies investigating the expression of Netrin‐1 and BCMA in mature human lymphoid malignancies are sparse [10, 11, 12, 13, 14, 15], and mostly focusing on a limited range of lymphoma subtypes or involving only a small number of cases. While Netrin‐1 is expressed by MM [12], mantle cell lymphomas (MCL) and diffuse large B‐cell lymphomas (DLBCL) [11], there is no currently available study regarding its expression in other lymphoma subtypes. BCMA is expressed in plasmablastic lymphomas (PBL) and MM [10, 15]; however, in large B‐cell lymphomas and Hodgkin lymphoma, study results are inconsistent [9]. Some findings indicate that BCMA expression is restricted to plasma cells within the tumor microenvironment, while others report BCMA labeling in Hodgkin cells in up to 80% of cases [10, 14, 16]. Additionally, most studies have been conducted using flow cytometry rather than immunohistochemistry (IHC), which may contribute to these discrepancies [10].

This study aimed to evaluate the expression of Netrin‐1 and BCMA in a large cohort of mature lymphoid neoplasms, to assess their potential as biomarkers or therapeutic targets in additional lymphoma subtypes.

## Materials and methods

### Selection of cases

In the present study, all cases of lymphomas investigated in the pathology department of the Hôpital Lyon Sud, Lyon, France, between January 2010 and August 2019, were retrospectively retrieved. All new diagnoses of lymphomas performed in France since 2010 were reviewed by expert pathologists (MD, JF, AT‐G) from the French Lymphopath Network [17] according to the 2022 World Health Organization (WHO) or the International Consensus Classification (ICC) [18, 19]. All cases with typical diagnoses of mature lymphoid neoplasm and sufficient material to perform tissue microarray were included in the analysis.

This study was conducted in accordance with the Declaration of Helsinki and the guidelines of the French Bioethics Law, with written informed consent obtained from all subjects or their legal guardians for diagnosis and the use of data for research purposes.

### Netrin‐1 and BCMA IHC

**Figure 1:**
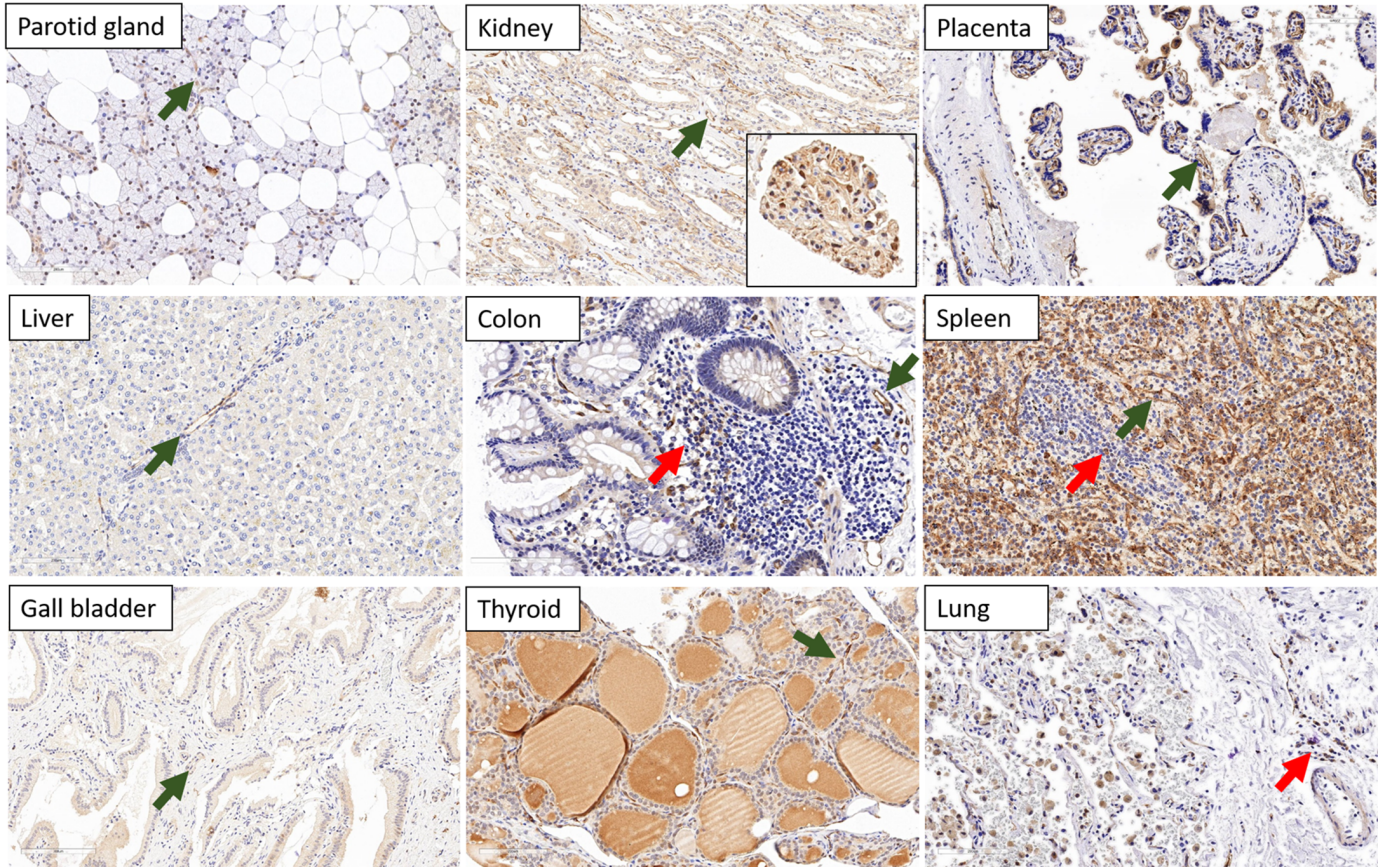
Immunohistochemical study using anti‐Netrin‐1 antibody in healthy tissues. No expression of Netrin‐1 was detected in the parotid gland, liver, gallbladder, renal medulla, thyroid (nonspecific staining in the colloid), placenta, spleen, or lung. In all these organs, internal positive controls were present, with staining observed in capillaries (green arrows) or normal plasma cells (red arrows). Netrin‐1 was expressed by the podocytes of the kidney glomerulus (inset in the image from the renal medulla).

**Figure 2:**
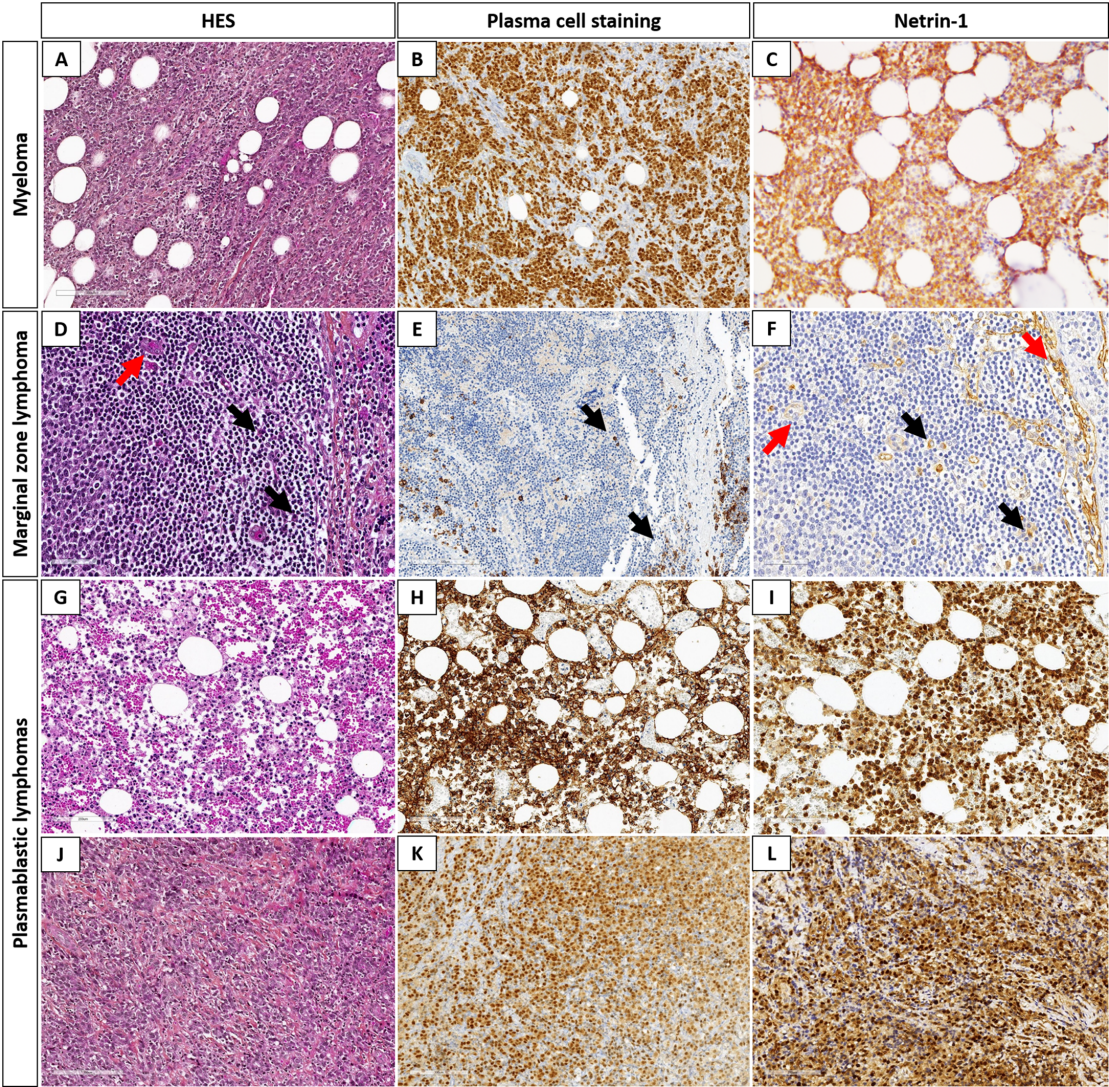
Immunohistochemical study using anti‐Netrin‐1 antibody. (A–C) Hematoxylin eosin saffron (HES) (×10), MUM1 (×10), and Netrin‐1 (×10) in myeloma. (D–F): HES (×10), CD38 (×10), and Netrin‐1 (×10) in a marginal zone lymphoma. Netrin‐1 displays staining in normal plasma cells (black arrows) and blood vessels (red arrows). (G–I) HES (×10), CD38 (×10), and Netrin‐1 (×10) in a plasmablastic lymphoma. In this case, Netrin‐1 displayed intermediate to strong staining of 40% of cells. (J–L) HES (×10), MUM1 (×10) and Netrin‐1 (×10) in a plasmablastic lymphoma. In this case, Netrin‐1 displayed intermediate to strong staining of 90% of cells.

**Figure 3:**
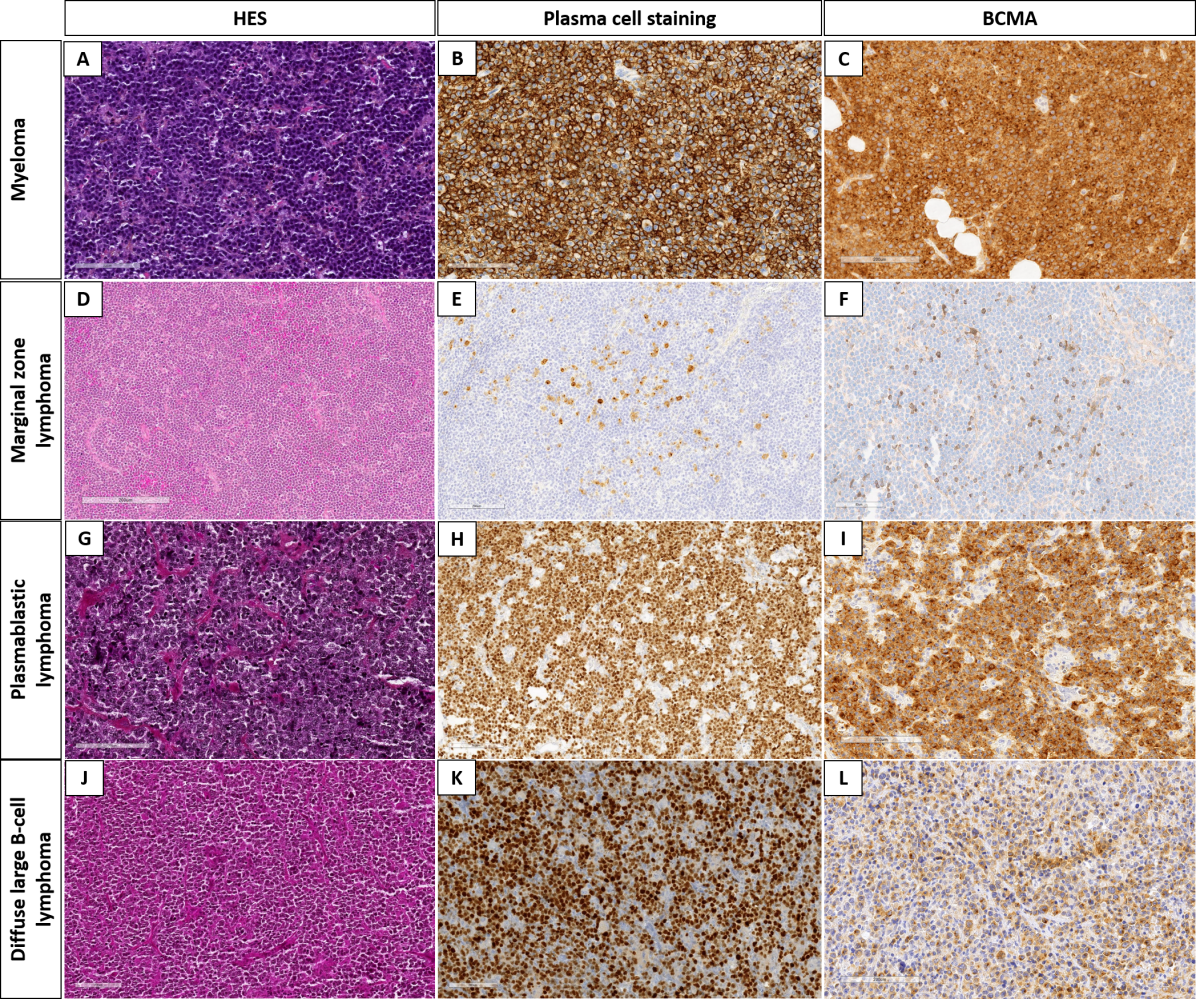
Immunohistochemical study with anti‐BCMA antibody. (A–C) HES (×10), CD38 (×10), and BCMA (×10) in myeloma. BCMA in myeloma displayed an intense membranous pattern associated with a cytoplasmic dot in the Golgi area. (D–F) HES (×10), CD38 (×10), and BCMA (×10) in a marginal zone lymphoma. BCMA stained only normal plasma cells. (G–I) HES (×10), CD38 (×10), and BCMA (×10) in a plasmablastic lymphoma. In this case, BCMA displayed intermediate to strong staining of 90% of cells. (J–L) HES (×10), MUM1 (×10) and BCMA (×10) in a diffuse large B‐cell lymphoma. BCMA displayed a weak or moderate staining in almost 30% of the cells.

In this study, four anti‐Netrin‐1 antibodies and two anti‐BCMA antibodies were assessed (supplementary material, Table S1) using healthy tissues (Figure 1 and supplementary material, Figure S1), tonsils, and myeloma cases as controls; Netrin‐1 (Figure 2) and BCMA (Figure 3) are known to be expressed in myelomas.

Sections were cut at 3 μm thickness from paraffin blocks, dried, de‐waxed, and rehydrated; slides were unmasked with Epitope Retrieval Solution 1 (Leica Biosystems, Wetzlar, Germany). The six antibodies were incubated with a commercially available detection kit (Bond Polymer Refine Detection, Leica Biosystems) in an automated immunostainer (BOND‐MAX, Leica Biosystems). Tests with different incubation times, pHs, or antibody dilutions were performed. The anti‐Netrin‐1 CPA2389 (Bioscience, Oxford, MS, USA, dilution 1/100) and BCMA D‐6, sc‐390147 (Santa Cruz Biotechnology, Dallas, TX, USA; dilution 1/100) antibodies were finally selected due to their lower background signal.

Two pathologists (MD, AT‐G) evaluated the expression of Netrin‐1 and BCMA in tumor cells through a double‐blind evaluation. Discordant cases were collegially reviewed (MD, JF, AT‐G). Results were assessed as a percentage of positive cells. The case was considered positive when more than 5% of tumor cells were positive. Data were expressed as count and percentage, and sensitivity as well as specificity were assessed with their 95% confidence intervals (95% CI).

## Results

The studied cohort included 361 cases, comprising 261 B‐cell lymphomas, 45 T‐cell lymphomas, and 55 classical Hodgkin lymphomas (cHL); B‐cell lymphomas comprised 79 follicular lymphomas (FL), 43 DLBCL, 40 nodal marginal zone lymphomas (NMZL), 35 chronic lymphocytic leukemias (CLL), 28 PBL, 28 MCL, and 8 lymphoplasmocytic lymphomas (LPL); T‐cell lymphomas comprised 24 angio‐immunoblastic T‐cell lymphomas (AITL), 12 peripheral T‐cell lymphomas NOS (PTCL‐NOS), and 9 anaplastic large cell lymphomas (ALCL), ALK‐positive; and cHL comprised 45 nodular sclerosis cases and 10 mixed‐cellularity cases.

On the tonsils used as internal control, normal plasma cells were positive using Netrin‐1 and BCMA antibodies (Figures 2 and 3). Blood vessels were also positive for Netrin‐1 (Figure 2F), as described in the literature data [20].

### B‐cell lymphomas (*n* = 261)

**Figure 4:**
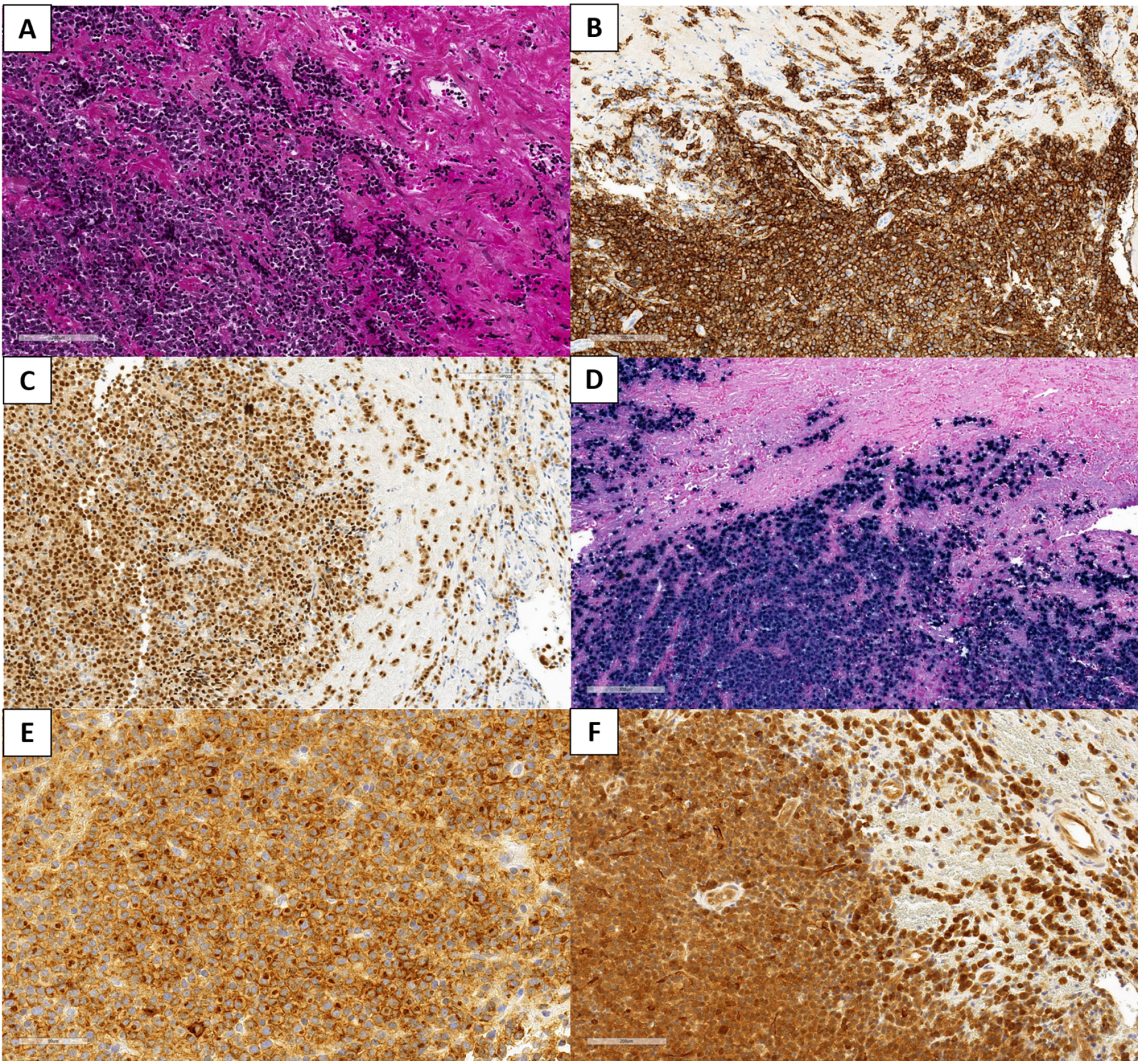
Netrin‐1 and BCMA immunostaining in plasmablastic lymphoma. (A) HES (×10) section of a plasmablastic lymphoma case. (B) CD38 (×10) and (C) MUM1 (×10): expression of plasma cell markers in tumoral cells, grouped in clusters or dispersed in fibrosis. (D) EBER (×10): *in situ* hybridization for Epstein–Barr virus (EBV) encoded small nuclear RNAs (EBERs) highlighting overexpression of EBV in more than 80% of cells. (E) BCMA (×20) displaying expression in most cells, comprising membrane staining associated with a cytoplasmic dot in the Golgi area. (F) Netrin‐1 (×20) displaying expression in most cells, with cytoplasmic staining.

Apart from PBL, subtypes of B‐cell lymphomas were negative using the anti‐Netrin‐1 antibody (Table 1); in lymphomas with plasma cell differentiation (NMZL and LPL), the antibody only stained plasma cells. Regarding PBL, the large majority were positive (27/28, 96%; Table 2) and exhibited a cytoplasmic staining pattern for Netrin‐1 (Figures 2 and 4). In most cases (21/27, 78%), more than 10% of tumor cells were positive, and in 11/27 (41%) PBL, more than 50% of tumor cells were positive. Considering all these results, the anti‐Netrin‐1 antibody displayed a sensitive [96%, 95% CI (82%; 100%)] and specific [100%, 95% CI (98%; 100%)] staining in PBL, as compared to other subtypes of B‐cell lymphomas.

**Table 1:** Immunohistochemistry results for Netrin‐1 in lymphomas

	Positive cases, *N* (%)	Negative cases, *N* (%)
B‐cell lymphomas (*N* = 261)	27 (10)	233 (89)
Follicular lymphomas (*N* = 79)	0 (0)	79 (100)
Diffuse large B‐cell lymphomas (*N* = 43)	0 (0)	43 (100)
Nodal marginal zone lymphomas (*N* = 40)	0 (0)	40 (100)
Chronic lymphocytic leukemia (*N* = 35)	0 (0)	35 (100)
Mantle cell lymphomas (*N* = 28)	0 (0)	28 (100)
Plasmablastic lymphomas (*N* = 28)*	27 (96)	0 (0)
Lymphoplasmacytic lymphomas (*N* = 8)	0 (0)	8 (100)
Classical Hodgkin lymphomas (*N* = 55)	8 (15)	47 (85)
Nodular sclerosis (*N* = 45)	4 (9)	41 (91)
Mixed cellularity (*N* = 10)	4 (40)	6 (60)
T‐cell lymphomas (*N* = 45)	0 (0)	45 (100)
Angio‐immunoblastic T‐cell lymphomas (*N* = 24)	0 (0)	24 (100)
Peripheral T‐cell lymphomas NOS (*N* = 12)	0 (0)	12 (100)
Anaplastic large cell lymphomas, ALK‐positive (*N* = 9)	0 (0)	9 (100)

* Among the 28 plasmablastic lymphomas, 1 was not evaluated since there was no staining for the positive control.

**Table 2:** Immunohistochemistry results for Netrin‐1 in plasmablastic lymphomas

	Plasmablastic lymphomas (*n* = 28)
Positive cases (≥5% of cells)	27 (96.4%)
5–9%	6 (21.4%)
10–50%	10 (35.7%)
≥50%	11 (39.3%)
Negative cases (<5% of cells)	1 (3.6%)

Most subtypes of B‐cell lymphoma were also not positive for BCMA (Table 3); in lymphomas with plasma cell differentiation (NMZL or LPL), the anti‐BCMA antibody only stained plasma cells. As for Netrin‐1, the large majority of PBL were positive for BCMA (17/20, 85%). Of note, among the 28 PBL, 8 could not be analyzed due to material exhaustion. In all positive PBL, BCMA staining was of a cytoplasmic pattern with a cytoplasmic dot in the Golgi area (Figure 4); the percentage of positive tumor cells ranged from 10% to 100%. In 53% of cases (9/17), more than 50% of the cells were positive. Altogether, these results highlight that the anti‐BCMA antibody displayed sensitive [85%, 95% CI (63%; 95%)] and specific [95%, 95% CI (91%; 97%)] staining in PBL. In addition, BCMA was also positive in DLBCL (8/43, 19%; Figure 3) and FL (4/79, 5%), with an intermediate expression level. Among positive cases of DLBCL, six (75%) were of non‐germinal center (GC) phenotype, and two (25%) were of GC phenotype.

**Table 3:** Immunohistochemistry results for BCMA in lymphomas

	Positive cases, *N* (%)	Negative cases, *N* (%)
B‐cell lymphomas (*N* = 253)	29 (11)	224 (89)
Follicular lymphomas (*N* = 79)	4 (5)	75 (95)
Diffuse large B‐cell lymphomas (*N* = 43)	8 (19)	35 (81)
Nodal marginal zone lymphomas (*N* = 40)	0 (0)	40 (100)
Chronic lymphocytic leukemia (*N* = 35)	0 (0)	35 (100)
Mantle cell lymphomas (*N* = 28)	0 (0)	28 (100)
Plasmablastic lymphomas (*N* = 20)*	17 (85)	3 (11)
Lymphoplasmacytic lymphomas (*N* = 8)	0 (0)	8 (100)
Classical Hodgkin lymphomas (*N* = 55)	0 (0)	55 (100)
Nodular sclerosis (*N* = 45)	0 (0)	45 (100)
Mixed cellularity (*N* = 10)	0 (0)	10 (100)
T‐cell lymphomas (*N* = 45)	0 (0)	45 (100)
Angio‐immunoblastic T‐cell lymphomas (*N* = 24)	0 (0)	24 (100)
Peripheral T‐cell lymphomas NOS (*N* = 12)	0 (0)	12 (100)
Anaplastic large cell lymphomas, ALK‐positive (*N* = 9)	0 (0)	9 (100)

* Among the 28 plasmablastic lymphomas, 8 could not be analyzed due to material exhaustion.

### T‐cell lymphoma (*n* = 45)

T‐cell lymphomas did not show any staining using the anti‐Netrin‐1 or the anti‐BCMA antibodies.

### Classical Hodgkin lymphoma (*n* = 55)

**Figure 5:**
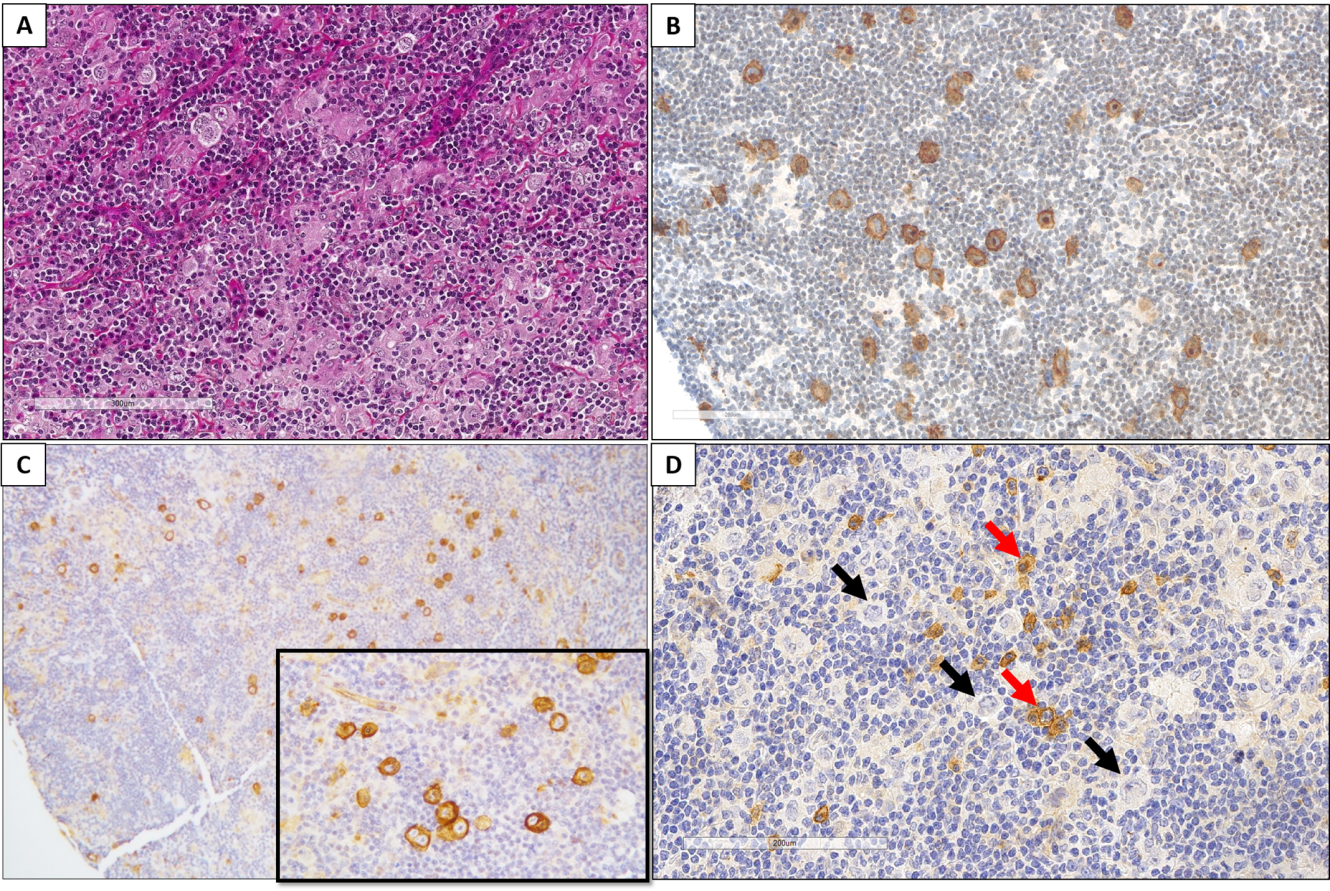
Netrin‐1 and BCMA immunostaining in classical Hodgkin lymphoma. (A) HES highlighting Reed‐Sternberg cells present in a mixed inflammatory background of eosinophils, neutrophils, mature lymphocytes, plasma cells, and histiocytes. (B) CD30 positivity in Reed‐Sternberg cells. (C) Netrin‐1 (×10) in a classical Hodgkin lymphoma case, highlighting membranous staining on the Reed‐Sternberg cells (see inset, ×40). (D) BCMA (×20) in a classical Hodgkin lymphoma case (black arrows: negative Reed‐Sternberg cells, red arrows: positive plasma cells).

Netrin‐1 stained Hodgkin/Reed‐Sternberg cells of cHL (8/55, 15%), including 4 nodular sclerosis cHL and 4 mixed cellularity cHL, with membranous staining on the Reed‐Sternberg cells (Figure 5C). Using the anti‐BCMA antibody, cHL cells showed no staining (Figure 5D).

## Discussion

The present study, which included a large cohort of different lymphomas, found expression of Netrin‐1 in most PBL and in few cHL. In addition, Netrin‐1 was also found in the plasma cells of MZL and LPL (B‐cell lymphomas with plasma cells differentiation). BCMA expression was found only in PBL, DLBCL, and FL subtypes. Of note, neither Netrin‐1 nor BCMA was found in T‐cell lymphomas.

This study is the first to describe Netrin‐1 expression in Hodgkin cells of cHL. Although sensitivity is weak, Netrin‐1 could be an interesting marker to differentiate cHL from other mature B‐cell lymphomas. Studies including nodular lymphocyte predominant Hodgkin lymphoma could reinforce the interest of Netrin‐1 as a specific biomarker of cHL. Conversely, there was no expression of BCMA in cHL herein, which is discordant with the data from the literature. Friedman *et al* highlighted BCMA expression in >5% of the tumor cells in 57% of cHL patient biopsies [16]. In the study by Chiu *et al*, BCMA transcript and protein were expressed in 80% of cHL cell lines [14]. However, in both Lee *et al* and Khattar *et al* studies, BCMA was negative in cHL [4, 21].

Regarding PBL, the observed BCMA positivity herein is consistent with the data reported in the literature [3, 15]. These results reinforce the interest in developing clinical trials testing BCMA monoclonal antibodies in PBL, which is known to have a particularly poor outcome (particularly in refractory cases with weak therapeutic arsenal). No optimal treatment has indeed been established for PBL, in which the prognosis remains poor, demonstrating the urgent need for the development of effective treatment alternatives to high‐dose chemotherapies. Interestingly, BCMA antibody‐drug conjugate, anti‐BCMA Chimeric Antigenic Receptor‐T cells (CAR‐T cells) therapy, and bispecific BCMA‐directed CD3 T‐cell engager have shown impressive results in MM [8, 19]. In this context, studying such target therapies in PBL will be interesting [13]. Moreover, while some studies have already highlighted a particular interest in studying Netrin‐1 in plasma cell neoplasms, particularly MM [12], the present study is the first to describe Netrin‐1 expression in PBL with sensitive and specific staining.

Additionally, the present study, conducted on a large cohort of B‐cell lymphomas, confirms the literature findings regarding BCMA positivity in DLBCL and FL [10]. However, the results for CLL differ significantly from previous studies, which reported a significantly higher BCMA mean fluorescence intensity on CLL cells compared to normal B cells using flow cytometry [22]. In contrast, studies on BCMA mRNA expression appear more aligned with our findings, showing an absence of expression in CLL when compared to normal plasma cells or MM cells [21, 23].

The present findings should however be interpreted with caution. For instance, herein, Netrin‐1 was not found in MCL and DLBCL, whereas Broutier *et al* reported Netrin‐1 expression in 55.6% of MCL and 71.4% of DLBCL. The difference between this protocol and the present one may explain this discrepancy. Broutier *et al* employed a distinct antibody (AF1109, R&D Systems, Minneapolis, MN, USA), corresponding to a polyclonal goat IgG, which is validated for Western blot and blockade of receptor‐ligand interaction in mouse samples [11]. While anti‐mouse antibodies can be utilized in IHC on human tissues, this approach may result in interference due to cross‐reactivity or the presence of human anti‐mouse antibodies, potentially causing false‐positive outcomes or elevated background noise. During our study's antibody evaluation phase, the antibody used by Broutier *et al* failed to stain the control tissues in our experiments and was consequently excluded from further analysis. Instead, we selected a rabbit polyclonal antibody, validated for both IHC and Western blotting in human specimens, which has also been employed in a recent investigation concerning endometrioid endometrial adenocarcinoma [24, 25]. However, since their findings were confirmed using reverse transcription polymerase chain reaction (RT‐PCR), the absence of Netrin‐1 expression in MCL and DLBCL herein may be related to its degradation. Future studies will focus on performing RT‐PCR as well as *in situ* hybridization to validate these preliminary results. Additionally, staining of a cell line and a CRISPR‐edited Netrin‐1/BCMA knockout of the same cell line could further validate the antigen specificity of the staining.

However, the results of the present study may be helpful for pathologists in establishing the differential diagnosis between PBL and other malignant B‐cell lymphomas, which can be difficult. The problem arises mainly between PBL and different subtypes of DLBCL, particularly in cases of immunoblastic cytology or Epstein–Barr virus (EBV)‐positive DLBCL [19]. DLBCL and PBL may indeed have similar clinical presentations. In most cases, PBL occurs in the context of immunodeficiency [due to human immunodeficiency virus (HIV), autoimmune diseases, or immunosuppressive treatments] and is associated with EBV infection. However, EBV‐positive DLBCLs or lymphoproliferative disorders also exist. The immunophenotype of PBL includes CD138, CD38, and MUM1 positivity, but some cases have an atypical presentation. In a series of 35 PBL, Montes‐Moreno *et al* illustrated that 43% expressed less than two plasma cell markers [26]. Moreover, only 37% showed surface expression of CD138 [26]. Pan–B‐cell markers (CD20, PAX‐5) are usually absent, but 1–2% of DLBCL can also lose the expression of CD20 [19, 26]. The details of the immunohistochemical characteristics of the 28 PBL analyzed in the present study are available in supplementary material, Table S2. In addition, NGS, widely used for lymphoma diagnosis, is not very useful in these lymphomas, with few specific mutations described [20, 21, 22]. These data highlight the difficulty of affirming a diagnosis of PBL rather than other subtypes of DLBCL. This illustrates the need to find new specific and sensitive markers of the plasma cell lineage and the potential interest in using both Netrin‐1 and BCMA IHC in daily practice. Of note, due to their expression in both PBL and myelomas, IHC with Netrin‐1 or BCMA is unlikely to assist in the challenging differential diagnosis between plasmablastic lymphoma and plasmablastic myeloma.

Regarding therapeutic opportunities, it is admitted that Netrin‐1 promotes tumor cell survival (Unc‐5 Netrin Receptor B) through interactions with receptors such as DCC and UNC5. Studies suggest that Netrin‐1 is overexpressed in certain lymphomas, such as DLBCL, potentially driving their aggressiveness and resistance to treatment [11]. Preclinical models showed that inhibiting Netrin‐1 can increase tumor cell apoptosis, making it a promising therapeutic target, with potential use in combination therapies such as chemotherapy or rituximab [8, 11]. However, further research is needed to confirm its clinical relevance in lymphomas.

In conclusion, the present study highlights both BCMA and Netrin‐1 expression in PBL, with sensitive and specific staining. It is also the first description of Netrin‐1 expression in Hodgkin cells of cHL. This underscores the potential of these molecules as diagnostic tools for pathologists, biomarkers, and therapeutic targets. Confirmation of these results by RT‐PCR could reinforce the interest in developing clinical trials targeting Netrin‐1 or BCMA in PBL and cHL.

## Author contributions statement

AT‐G, CD, and HG conceived and planned the experiments. MDo, MDe, EC‐P, T‐T‐TN and SD carried out the experiments. JF and AT contributed to sample selection. MDo, T‐T‐TN and SD contributed to the interpretation of the results. MDo wrote the manuscript. All authors provided critical feedback and helped shape the research, analysis and manuscript.

## Supporting information


**Figure S1.** Netrin‐1 expression in endometrioid endometrial adenocarcinoma and healthy endometrium
**Table S1.** Antibodies tested in the present study
**Table S2.** Immunohistochemical characteristics of the 28 analyzed plasmablastic lymphomas

## Data Availability

The authors confirm that the data supporting the findings of this study are available within the article or its supplementary data.
