# Supplementary material for: Netrin‐1 and B‐cell maturation antigen expression in a large cohort of 361 lymphomas: sensitive and specific staining in plasmablastic lymphomas, and therapeutic perspectives
Source: J Pathol Clin Res. 2025 Apr 15;11(3):e70027. doi: 10.1002/2056-4538.70027 (PMC12000542; doi:10.1002/2056-4538.70027)
Supplement: Supplementary file 1 — Figure S1. Netrin‐1 expression in endometrioid endometrial adenocarcinoma and healthy endometrium Table S1. Antibodies tested in the present study Table S2. Immunohistochemical characteristics of the 28 analyzed plasmablastic lymphomas [file CJP2-11-e70027-s001.pdf]

Netrin-1 and B-cell maturation antigen expression in a large cohort of 361 lymphomas: sensitive and specific staining in plasmablastic lymphomas, and therapeutic perspectives

M Donzel *et al*., *J Pathol Clin Res*, https://doi.org/10.1002/2056-4538.70027

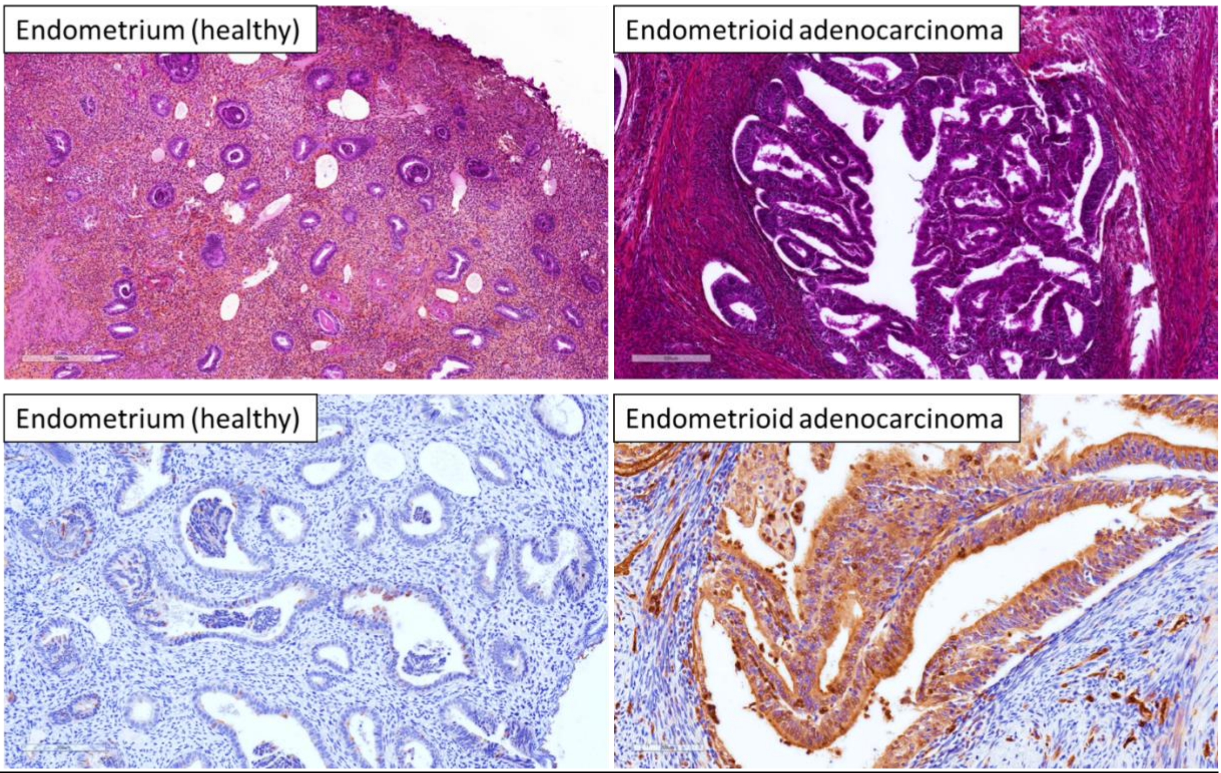

Figure S1. Netrin-1 expression in endometrioid endometrial adenocarcinoma and healthy endometrium.

Figure illustrating Netrin-1 positivity in an additional positive control (besides myelomas) in which Netrin-1 expression is admitted: an endometrioid adenocarcinoma [24]. We compared these results to Netrin-1 expression in a healthy tissue (an endometrium in the proliferative phase). Netrin-1 expression was observed in carcinoma but not in healthy endometrium epithelial cells (internal positive controls: capillaries and normal plasma cells), as described in literature data [25].

Table S1. Antibodies tested in the present study

Antibodies	Provider	Reference	Source
**Netrin-1**	Abcam	ab39370	Chicken
**Netrin-1**	R&D	AF1109	Goat
**Netrin-1**	Bioscience	CPA2389	Rabbit
**Netrin-1**	Abcam	Ab126729	Rabbit
**BCMA**	R&D	MAB10762	Mouse
**BCMA (D-6)**	Santa Cruz Biotechnology	sc-390147	Mouse

Table S2. Immunohistochemical characteristics of the 28 analyzed plasmablastic lymphomas.

	Immunosuppressive disorder	*EBV-EBER*	*CD20*	*%CD20*	*PAX5*	*CD79a*	*%CD79A*	*CD38*	*%CD38*	*CD138*	*%CD138*	*MUM1*	*%MUM1*	*EMA*	*%EMA*	*Light chain restriction*	*CD56*	*CCND1*	*HHV8*	*CMYC*	*%CMYC*	*FISH CMYC*	*ALK*	*Ki67*	*Netrin-1*	*BCMA*
Case n°01	NA	0	0		0	0		1	90%	0		1	80%	NA		NA	NA	NA	0	NA		NA	0	90%	70%	0
Case n°02	NA	1	1	20%	0	1	20%	1	60%	0		1	80%	0		no	NA	NA	0	NA		NA	0	70%	20%	10%
Case n°03	NA	0	0		0	NA		1	10%	1	50%	1	40%	1	40%	NA	0	0	0	0		negative	0	80%	10%	0
Case n°04	YES	1	1	25%	0	1	90%	1	10%	1	10%	1	80%	NA		NA	NA	NA	0	NA		NA	0	70%	10%	NA
Case n°05	NO	0	0		NA	0		1	10%	1	20%	1	70%	0		NA	0	0	0	NA		NA	0	70%	40%	NA
Case n°06	YES	1	0		0	0		1	20%	1	80%	1	80%	0		kappa	NA	NA	0	NA		NA	0	80%	40%	80%
Case n°07	YES	1	0		0	0		1	90%	1	80%	1	70%	0		lambda	NA	NA	0	1	90%	90%	NA	90%	10%	20%
Case n°08	NA	0	0		NA	0		0		1	80%	1	60%	0		NA	NA	NA	0	NA		NA	0	35%	NA	20%
Case n°09	YES	1	0		0	0		1	50%	1	80%	1	90%	NA		NA	1	0	0	1	90%	negative	NA	90%	15%	NA
Case n°10	NO	1	0		0	1	20%	1	10%	0		1	100%	0		NA	0	0	0	1	50%	negative	0	90%	80%	30%
Case n°11	NO	1	0		NA	0		1	50%	1	70%	1	90%	NA		NA	0	0	0	1	90%	80%	0	90%	20%	80%
Case n°12	NA	1	0		0	0		1	10%	1	20%	1	20%	1	100%	no	NA	NA	0	0		90%	0	95%	10%	0
Case n°13	NA	1	0		NA	0		1	60%	1	100%	1	70%	0		NA	1	0	0	1	60%	90%	NA	85%	70%	95%
Case n°14	NA	0	0		NA	NA		1	80%	1	NA	1	90%	NA		kappa	NA	NA	0	1	10%	negative	0	40%	20%	NA
Case n°15	NA	1	0		NA	1	10%	1	90%	1	40%	1	60%	1	10%	NA	0	NA	0	0		negative	0	90%	20%	20%
Case n°16	YES	1	0		NA	0		0		1	95%	1	95%	NA		NA	NA	NA	0	NA		NA	NA	85%	10%	40%
Case n°17	NA	0	0		NA	1	20%	1	95%	1	NA	1	NA	1	NA	NA	0	0	0	NA		NA	NA	65%	80%	60%
Case n°18	NA	0	0		0	0		1	95%	1	95%	1	90%	1	100%	kappa	1	NA	0	1	50%	NA	NA	90%	10%	NA
Case n°19	NA	1	0		0	0		1	90%	0		1	50%	0		no	0	NA	0	1	60%	100%	0	90%	20%	10%
Case n°20	YES	1	0		0	0		0		0		1	90%	1	60%	kappa	0	NA	0	NA		NA	0	95%	95%	NA
Case n°21	NA	1	0		0	1	20%	1	90%	0		1	60%	0		kappa	0	NA	0	NA		NA	NA	15%	75%	80%
Case n°22	NA	0	0		0	0		1	95%	1	70%	1	NA	0		kappa	0	NA	0	1	80%	NA	0	70%	60%	NA
Case n°23	NA	1	0		0	NA		0		1	90%	1	80%	0		NA	0	0	0	1	70%	negative	0	70%	70%	15%
Case n°24	YES	1	0		0	0		1	95%	1	60%	1	80%	0		kappa	1	0	0	1	20%	negative	0	90%	60%	60%
Case n°25	NA	1	0		0	0		1		1	NA	1	NA	0		NA	0	0	0	0		negative	0	60%	40%	NA
Case n°26	NA	1	0		NA	1	30%	1	95%	0		1	90%	1	NA	kappa	0	NA	0	0		NA	0	80%	80%	95%
Case n°27	NA	0	0		0	1	5%	1	95%	1	10%	1	NA	0		lambda	0	0	0	1	40%	NA	0	30%	70%	60%
Case n°28	YES	1	0		0	0		1	60%	0		1	80%	1	10%	no	0	0	0	1	90%	90%	0	90%	30%	95%

EBV-EBERs : Epstein-Barr virus-encoded small RNAs, FISH : Fluorescence in situ hybridization, NA : not available
